# Preadmission course and management of severe pediatric group A streptococcal infections during the 2022–2023 outbreak: a single-center experience

**DOI:** 10.1007/s15010-024-02198-w

**Published:** 2024-03-01

**Authors:** Nina Schöbi, Andrea Duppenthaler, Matthias Horn, Andreas Bartenstein, Kristina Keitel, Matthias V. Kopp, Philipp Agyeman, Christoph Aebi

**Affiliations:** 1 Division of Pediatric Infectious Disease, Department of Pediatrics, Bern University Hospital, Inselspital, University of Bern, CH-3010 Bern, Switzerland; 2 Department of Pediatric Surgery, Bern University Hospital, Inselspital, University of Bern, Bern, Switzerland; 3 Pediatric Emergency Center, Department of Pediatrics, Bern University Hospital, Inselspital, University of Bern, Bern, Switzerland; 4 Airway Research Center North (ARCN), Member of the German Lung Research Center (DZL), University of Lübeck, Lübeck, Germany

**Keywords:** *Streptococcus pyogenes*, Invasive Group A Streptococcus, iGAS, Child, Outbreak, Guidelines

## Abstract

**Purpose:**

The massive increase of infections with Group A Streptococcus (GAS) in 2022–2023 coincided in Switzerland with a change of the recommendations for the management of GAS pharyngitis. Therefore, the objective of the present study was to investigate whether the clinical manifestations and management before hospitalization for GAS infection differed in 2022–2023 compared with 2013–2022.

**Methods:**

Retrospective study of GAS infections requiring hospitalization in patients below 16 years. Preadmission illness (modified McIsaac score), oral antibiotic use, and outcome in 2022–2023 were compared with 2013–2022. Time series were compared with surveillance data for respiratory viruses.

**Results:**

In 2022–2023, the median modified McIsaac score was lower (2 [IQR 2–3] vs. 3 [IQR 2–4], *p* =  < 0.0001) and the duration of preadmission illness was longer (4 days [3–7] vs. 3 [2–6], *p* = 0.004) than in 2013–2022. In both periods, withholding of preadmission oral antibiotics despite a modified McIsaac score ≥ 3 (12% vs. 18%, n.s.) or ≥ 4 (2.4% vs. 10.0%, *p* = 0.027) was rare. Respiratory disease, skeletal/muscle infection, and invasive GAS disease were significantly more frequent in 2022–2023, but there were no differences in clinical outcome. The time course of GAS cases in 2022–2023 coincided with the activity of influenza A/B.

**Conclusion:**

We found no evidence supporting the hypothesis that the 2022–2023 GAS outbreak was associated with a change in preadmission management possibly induced by the new recommendation for GAS pharyngitis. However, clinical manifestations before admission and comparative examination of time-series strongly suggest that viral co-circulation played an important role in this outbreak.

**Supplementary Information:**

The online version contains supplementary material available at 10.1007/s15010-024-02198-w.

## Introduction

A major increase in Group A Streptococcus (*Streptococcus pyogenes*) infections (GAS) in children and adolescents was observed in several European countries during the winter of 2022/2023 and included an increase in invasive GAS disease (iGAS) [1–5]. The exact cause of this resurgence is currently unknown and likely multifactorial. While lowered population immunity in the aftermath of the COVID-19 pandemic is a possible cause, genomic surveillance analyses from several European countries indicate that newly circulating clones of, e.g., *emm1* and *emm4* genotypes, may also play a role [6–8]. An association with the exceptionally severe 2022/2023 Respiratory Syncytial Virus (RSV) season has also been suggested [1], but in some countries including Switzerland, the activities of GAS and RSV were not closely related in time [8].

It is unclear whether changes in the management strategy of GAS pharyngitis have contributed to these high case numbers. In several countries, antibiotic therapy for uncomplicated GAS pharyngitis is no longer generally recommended, but considered optional given the only moderate effect on relief of acute, self-limited symptoms [9], and the extremely low incidence of acute rheumatic fever [10–12]. In Switzerland, this new policy was recommended in 2019 [13]. Primary care providers in particular have now questioned whether this change has favored the 2022–2023 resurgence of severe GAS disease. That such an effect did not become apparent until 3 years after the new recommendations had been issued, might be explained by the intervening COVID-19 pandemic, as public health measures designed to curb SARS-CoV-2 also suppressed the circulation of many other pathogens including GAS [1, 2, 4, 6, 14].

To address this question, we set out to examine the prehospitalization period of severe GAS infections in children and adolescents occurring during the massive surge of GAS disease in 2022–2023 in comparison to 2013–2022, when GAS pharyngitis was generally recommended to be treated with antibiotics. Our hypothesis was that a greater proportion of patients admitted in 2022–2023 had a preadmission illness compatible with GAS pharyngitis and did not receive an oral antibiotic than in earlier years. Thus, main outcome variables were the presence or absence of clinical manifestations consistent with GAS pharyngitis, the duration of illness before hospital admission, and the outpatient use of oral antibiotics.

## Methods

### Setting and case catchment

**Fig. 1:**
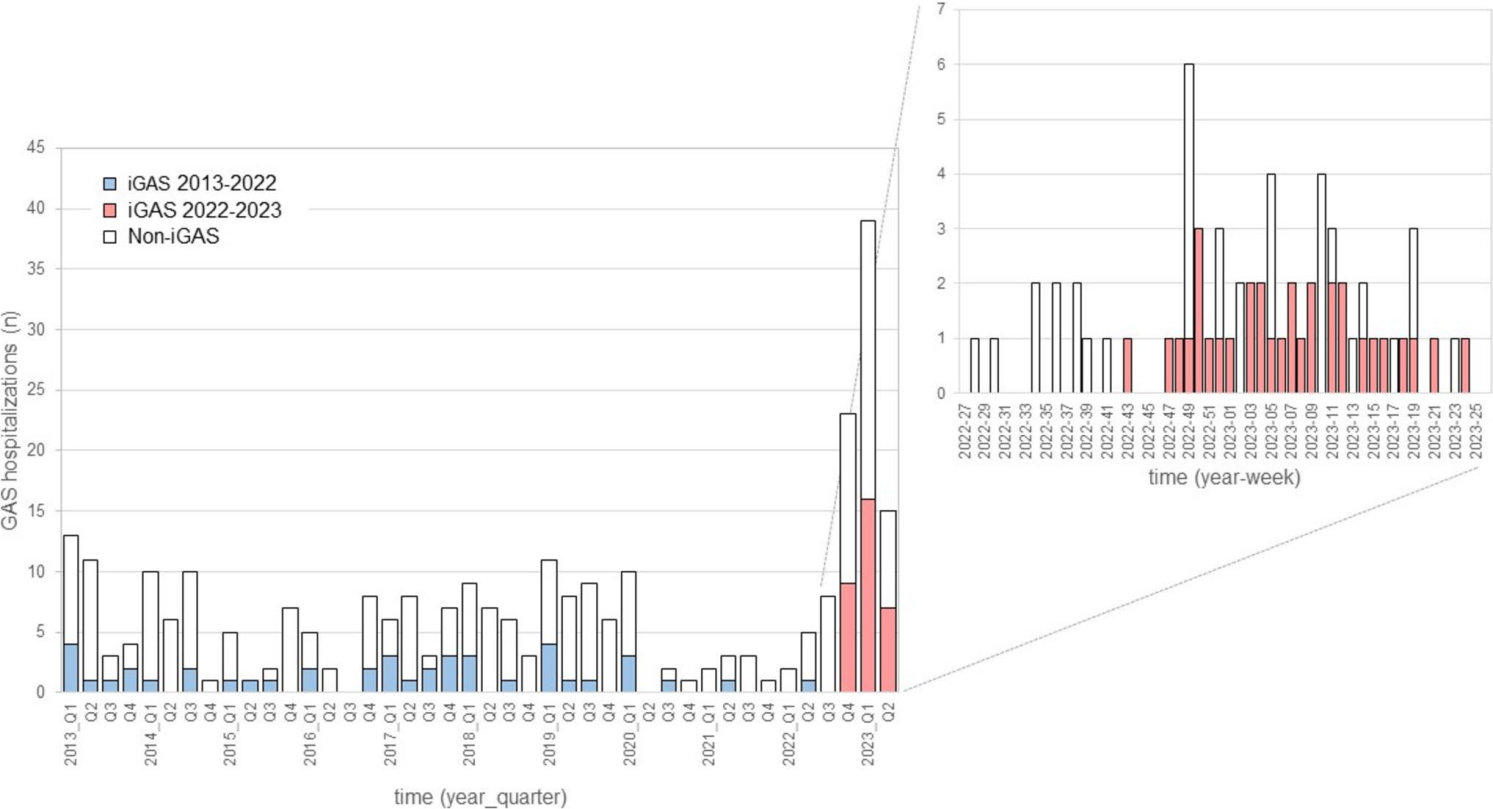
Hospitalizations because of infection with Group A Streptococcus (GAS) among children and adolescents aged < 16 years of age—Bern, Switzerland, January 2013–June 2023. Blue/red columns: iGAS cases; white columns, non-iGAS cases

This is a single-center study including all patients admitted to the Departments of Pediatrics and Pediatric Surgery, Bern University Hospital, Inselspital Bern, because of an acute, microbiologically confirmed infection with GAS between 1 January 2013 and 30 June 2023. Cases were identified retrospectively until 30 November 2022 and prospectively thereafter. Retrospective case identification consisted of (1) text-searching the electronic medical records for cases, in which the discharge summary listed a GAS infection as a main diagnosis, and (2) searching the clinical microbiology database for all GAS-positive results (culture and/or PCR) from specimens other than throat swabs. Prospective case identification consisted of (1) microbiological results positive for GAS (culture and/or PCR) from specimens other than throat swabs or (2) clinical presentation of toxic shock syndrome and or necrotizing fasciitis and GAS isolation from any site. Cases were included, if a patient was younger than 16.0 years and stayed as an in-patient for at least one night. Parental refusal to participate in this study was the only exclusion criterion. For each patient (identified retrospectively or prospectively), the data listed in Table 1 were extracted from the medical record. We then compared the case cohort admitted between 1 July 2022 and 30 June 2023 with the cohort admitted between 1 January 2013 and 30 June 2022. Even though the new GAS pharyngitis recommendations were issued in 2019 [13], we arbitrarily set the cut-off date for the two cohorts to 1 July 2022. This was based on the fact that there was little GAS activity during the first 2 years of the COVID-19 pandemic (Fig. 1) and our assumption that the adoption of the new recommendations by the primary care community would be a gradual process extending over several years.

**Table 1:** Clinical characteristics of patients hospitalized because of a GAS infection between 2013 and 2023

	Observation period					
	2022–2023	2013–2022	2013–2023			
Variable	value (%)	value (%)	value (%)	*p* value*	OR (95% CI)	aOR (95% CI)**
Demography						
Patients, *n*	85	200	285			
Male sex	55 (65)	122 (61)	177 (62)	0.554	1.17 (0.69–1.99)	
Age, median [IQR]	4.8 [2.4–8.1]	5.7 [3.2–9.4]	5.5 [ 2.9–8.1]	0.105	–	
Age ≥ 3 years	59 (69)	155 (78)	214 (75)	0.148	0.66 (0.37–1.16)	
Preadmission course						
Days from onset of symptoms to admission, median [IQR]	4 (3–7)	3 (2–6)	4 (2–7)	**0.004**	–	**1.07 (1.00–1.14)**
Physician visit(s) ≥ 1 day before admission	29 (34)	71 (36)	100 (35)	0.823	0.94 (0.55–1.60)	
Antimicrobial therapy initiated ≥ 1 day before admission	6 (7)	25 (13)	31 (11)	0.177	0.53 (0.21–1.35)	
Symptoms and signs						
Fever	70 (82)	167 (84)	237 (83)	0.806	0.92 (0.47–1.80)	
Sore throat	27 (32)	92 (46)	119 (42)	**0.026**	0.55 (0.32–0.94)	0.92 (0.46–1.85)
Cervical lymphadenopathy	15 (18)	88 (44)	103 (36)	** < 0.0001**	0.27 (0.15–0.51)	**0.28 (0.13–0.60)**
Nasal symptoms	22 (26)	32 16)	54 (19)	0.052	1.83 (0.99–3.39)	
Cough	29 (34)	23 (12)	52 (18)	** < 0.0001**	3.99 (2.13–7.44)	**3.48 (1.76–6.90)**
Vomiting	16 (19)	23 (12)	39 (14)	0.100	1.78 (0.89–3.58)	
Abdominal pain	6 (7)	10 (5)	16 (6)	0.575	1.44 (0.51–4.11)	
Scarlet fever-like rash	16 (19)	21 (11)	37 (13)	0.060	1.98 (0.97–4.01)	
Modified McIsaac Score ≥ 2 points	75 (88)	193 (97)	268 (94)	**0.007**	0.27 (0.10–0.74)	***
Modified McIsaac Score ≥ 3 points	45 (53)	149 (75)	194 (68)	**0.004**	0.39 (0.23–0.66)	***
Modified McIsaac Score ≥ 4 points	17 (20)	90 (45)	107 (38)	** < 0.0001**	0.31 (0.17–0.56)	***
Modified McIsaac Score, median [IQR]	3 (2, 3)	3 (2–4)	3 (2–4)	** < 0.0001**		***
Preadmission illness compatible with scarlet fever	16 (19)	20 (10)	36 (13)	**0.040**	2.09 (1.02–4.26)	0.79 (0.31–1.87)
Recent/concomitant varicella	8 (9)	9 (5)	17 (6)	0.109	2.20 (0.82–5.92)	
Diagnosis (primary organ system affected)						
Head, eye, ear, nose, throat	45 (53)	123 (62)	168 (59)	0.179	0.70 (0.42–1.18)	
Skin and soft tissue	14 (16)	51 (26)	65 (23)	0.097	0.58 (0.30–1.11)	
Respiratory tract	13 (15)	12 (6)	25 (9)	**0.011**	2.83 (1.23–6.49)	2.20 (0.77–6.35)
Skeletal and muscle systems	10 (12)	6 (3)	16 (6)	**0.005**	4.31 (1.51–12.28)	**3.57 (1.02–13.46**
Systemic onset	3 (4)	6 (3)	9 (3)	1.000	1.18 (0.29–4.84)	
Central nervous system	0	2 (1)	2 (1)	–	–	
Outcome						
Hospital stay, days [IQR]	5 (3–7)	5 (3–7)	5 (3–7)	0.363		
iGAS	32 (38)	43 (22)	75 (26)	**0.005**	2.20 (1.27–3.83)	0.93 (0.41–2.02)
Surgical intervention(s)	63 (74)	140 (70)	203 (71)	0.484	1.23 (0.69–2.17)	
Septic/toxic shock	8 (9)	18 (9)	26 (9)	0.920	1.05 (0.44–2.52)	
Intensive Care Unit (ICU) stay	13 (15)	29 (15)	42 (15)	0.862	1.06 (0.52–2.17)	
Mechanical ventilation	6 (7)	14 (7)	20 (7)	1.000	1.01 (0.37–2.72)	
Death	0	1 (0.5)	1 (0.4)	–	–	

*univariate analysis using *χ*^2^ test or Fisher's exact test (frequency data) or Mann–Whitney *U* test (ordinal data)

** aOR, adjusted odds ratio (multivariate logistic regression model including all variables that were significantly different in univariate analysis)

***not included in the multivariate analysis, because these scores were derivatives of the symptoms and signs included individually in the analysis

### Definitions

Invasive GAS infection (iGAS) was present when GAS was identified by culture or PCR in a specimen obtained from a normally sterile body site or when GAS was isolated from a surface culture and accompanied by necrotizing fasciitis and/or streptococcal toxic shock syndrome. Complicated eye and ear–nose–throat (ENT) infections with GAS being recovered surgically from the primary infection site only (e.g., orbital subperiosteal abscess) were not designated iGAS [15].

To assess which patients initially presented with a clinical pattern consistent with GAS pharyngitis, we adapted the sore throat score devised by McIsaac et al. [16] for the purpose of this study. The modification was necessary, because we extracted the patients’ symptoms and signs from the routine patient records. It is detailed in Table S1 of the supplementary data file. Scores ≥ 3 or ≥ 4, respectively, were considered “positive” in subsequent analyses, i.e., were defined to indicate clinical manifestations compatible with GAS pharyngitis during the preadmission period of the patients’ acute illness.

Clinical illness compatible with scarlet fever was defined as the presence of fever and an acute-onset generalized rash noted by a physician to be consistent with scarlatina.

### Analysis of preadmission events

To test the hypothesis described above, we recorded the preadmission events in each case using a standardized event sequence. It consisted of the occurrence or non-occurrence of three consecutive events: (1) physician visit(s) ≥ 1 day before admission [YES/NO], (2) clinical manifestations consistent with GAS pharyngitis (modified McIsaac Score positive) [YES/NO], and (3) an antimicrobial prescription was issued [YES/NO]. The endpoint was defined as “no antibiotic prescription issued”, because this sequence would identify patients having potentially benefitted from outpatient antibiotic therapy.

### Viral surveillance data

At our institution, viral studies are obtained as part of the routine microbial surveillance program from nasopharyngeal samples from all patients hospitalized with an acute lower respiratory tract infection. Each sample is tested for RSV, influenza A and B, parainfluenza 1–3, human metapneumovirus (hMPV), rhinovirus/enterovirus, and adenovirus using a Direct Immunofluorescence Antibody Panel (DFA) [17] and, for SARS-CoV-2, using quantitative RT-PCR. These surveillance data are publicly available at http://www.kinderklinik.insel.ch/fileadmin/Kinderklinik/Dokumente/Aktuelles/rsv-covid_dashboard.html.

### Statistical analysis and software

Data are presented as frequencies and proportions for categorical variables and as medians and interquartile ranges (IQR) for continuous variables. Categorical variables were compared with the χ2 test or Fisher’s exact test. Continuous variables were compared with the Mann–Whitney U test. P values below 0.05 were considered statistically significant. Odds ratios (OR) were adjusted by a multivariate logistic regression model that included all variables with a P value < 0.05 in univariate analyses. GraphPad Prism version 10.0.0 for Windows (GraphPad Software, Boston, Massachusetts USA, www.graphpad.com) or VassarStats (http://vassarstats.net/) was used for calculations.

## Results

### Epidemiology

Retrospective case search and prospective surveillance yielded a total of 294 GAS hospitalizations. Nine cases were removed from the analysis because of lack of parental consent. Figure 1 depicts the occurrence of cases over time during the entire study period. The top right inset illustrates the sharp increase in cases in early December 2022, followed by a steady decline until May 2023.

### Demographics, main diagnoses, and outcome until discharge

Clinical data of 285 cases occurring in 284 patients (one patient suffered from two independent episodes three years apart) are shown in Table 1. Male patients accounted for 62% of cases. The median age at admission was 5.5 years (IQR 2.9–8.1). While children below 3 years of age were overrepresented in 2022–2023, there was no significant difference in age at presentation between the two periods (Table 1 and Figure S1).

Comparing the main organ systems affected by GAS revealed that respiratory disease (almost exclusively pleural empyema) and skeletal and muscle disease occurred more frequently in 2022–2023, and that a greater proportion of cases were iGAS than in previous years (Table 1). However, this did not translate to worse outcome judged by the length of hospital stay, need for ICU admission, septic/toxic shock, and need for surgical interventions. Suppurative GAS disease located in the head and neck area occurred in similar proportions overall. While peritonsillar, para- and retropharyngeal abscesses (quinsy) were somewhat less common in 2022–2023, mastoiditis was more common than in 2013–2022. A detailed list of the diagnoses of all cases is provided in Table S2 with the numbers of cases fulfilling the aforementioned definition of iGAS infection in brackets (supplementary data file).

### Preadmission course of illness

Table 1 lists symptoms and signs commonly considered when GAS pharyngitis is suspected. Cases occurring in the 2022–2023 season presented significantly more often with cough, a scarlet fever-like illness, and a non-significant trend for nasal symptoms, while sore throat and cervical lymphadenitis were reported significantly less frequently than in previous years. These findings translated into modified McIsaac scores in 2022–2023 that were significantly lower than in hospitalized GAS cases in 2013–2022 (Figure S2, panel A). These data are also shown for patients above 3 years of age only (Figure S2, panel B), because the original McIsaac score was developed and validated for this age group only. In addition, we noted that the time interval from the reported onset of symptoms to hospital admission was significantly longer in 2022–2023 (supplementary date, Figure S3).

Multiple logistic regression of all retrieved clinical data revealed that the delay from the reported onset of symptoms to admission, the presence of cough and absence of cervical lymphadenitis, and skeletal/muscular disease were significantly associated with the GAS outbreak in 2022–2023 compared with the previous decade of observation.

### Preadmission antimicrobial therapy

**Fig. 2:**
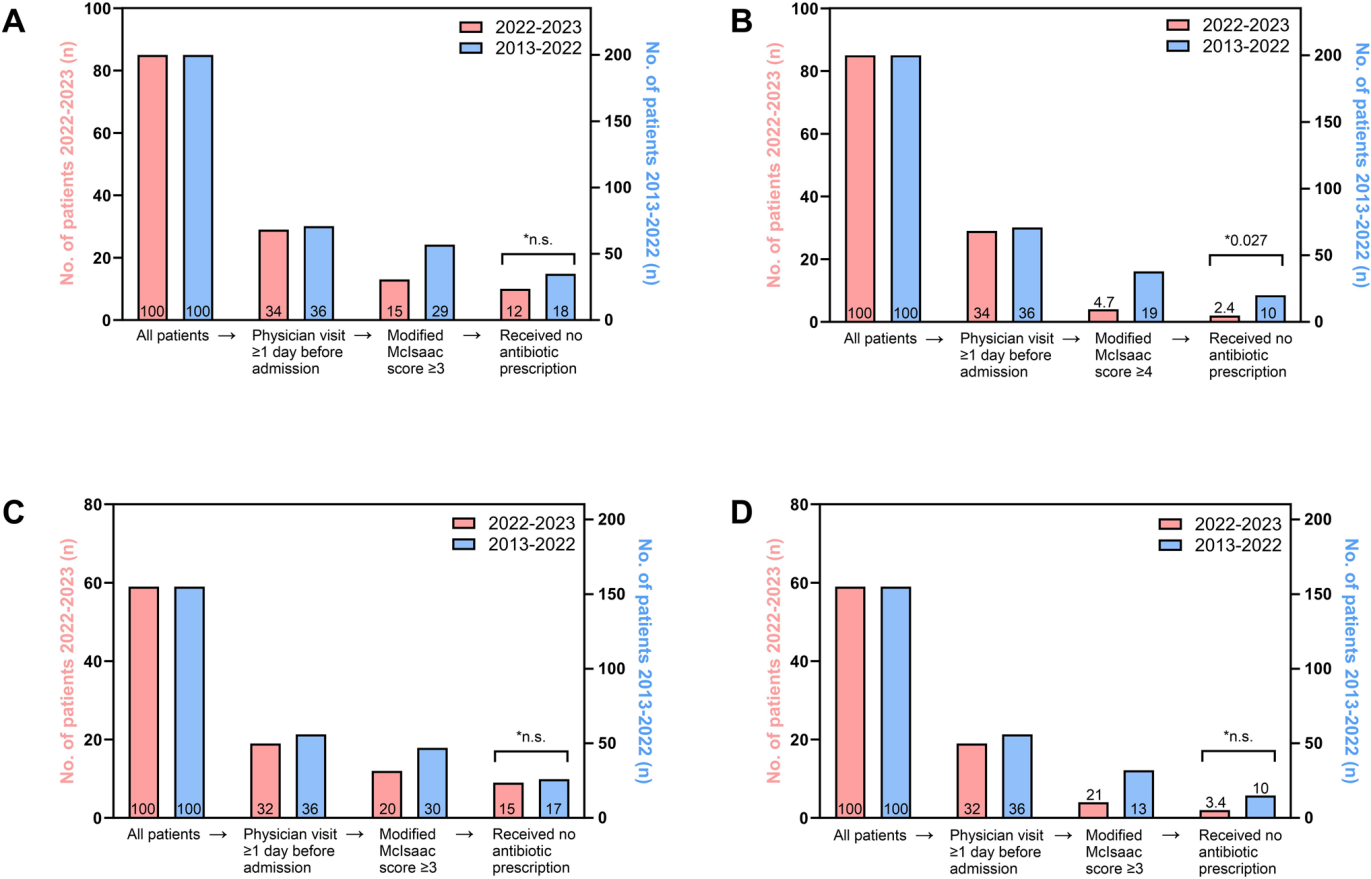
Preadmission event sequence (all patients → who had a physician visit ≥ 1 day before admission → and had a modified McIsaac score ≥ 3 [panel **A**] or ≥ 4 [panel **B**] → and received no antibiotic prescription) of patients hospitalized because of a GAS infection in 2022–2023 (red bars, left y-axis) or 2013–2022 (blue bars, right y-axis). The figures inside the bars represent the proportion (%) of all patients in the respective time period. The panels **C** (score ≥ 3) and **D** (score ≥ 4) illustrate the event sequences for patients 3 years of age and older only. *χ^2^ test

Overall, 11% of patients received a prescription for preadmission antimicrobial therapy ≥ 1 day before admission (Table 1). Among those who claimed a preadmission physician visit in 2022–2023, this rate was 21%. In 2013–2022, this proportion was 34% (OR 0.48; 95% CI 0.17–1.33; *p* = 0.233). Figure 2 illustrates the preadmission event sequences for patients in both study periods who claimed outpatient physician visits ≥ 1 day before admission, had a modified McIsaac Scores of ≥ 3 or ≥ 4 points, respectively, and were not prescribed an oral antibiotic (Fig. 2, panels A and B). For instance, using a score of ≥ 4 points, in 2022–2023 and 2013–2023, respectively, two (2.4%) and 20 patients (10%) (OR 0.22; 95% CI 0.05–0.95, *p* = 0.027) did not receive an antibiotic prescription despite the presence of symptoms and signs that were compatible with GAS pharyngitis (Fig. 2, panel B). The event sequences for the subgroup of patients above 3 years of age only are also shown (Fig. 2, panels C and D) and yielded similar results.

### Time series of hospitalizations with GAS and respiratory viruses

**Fig. 3:**
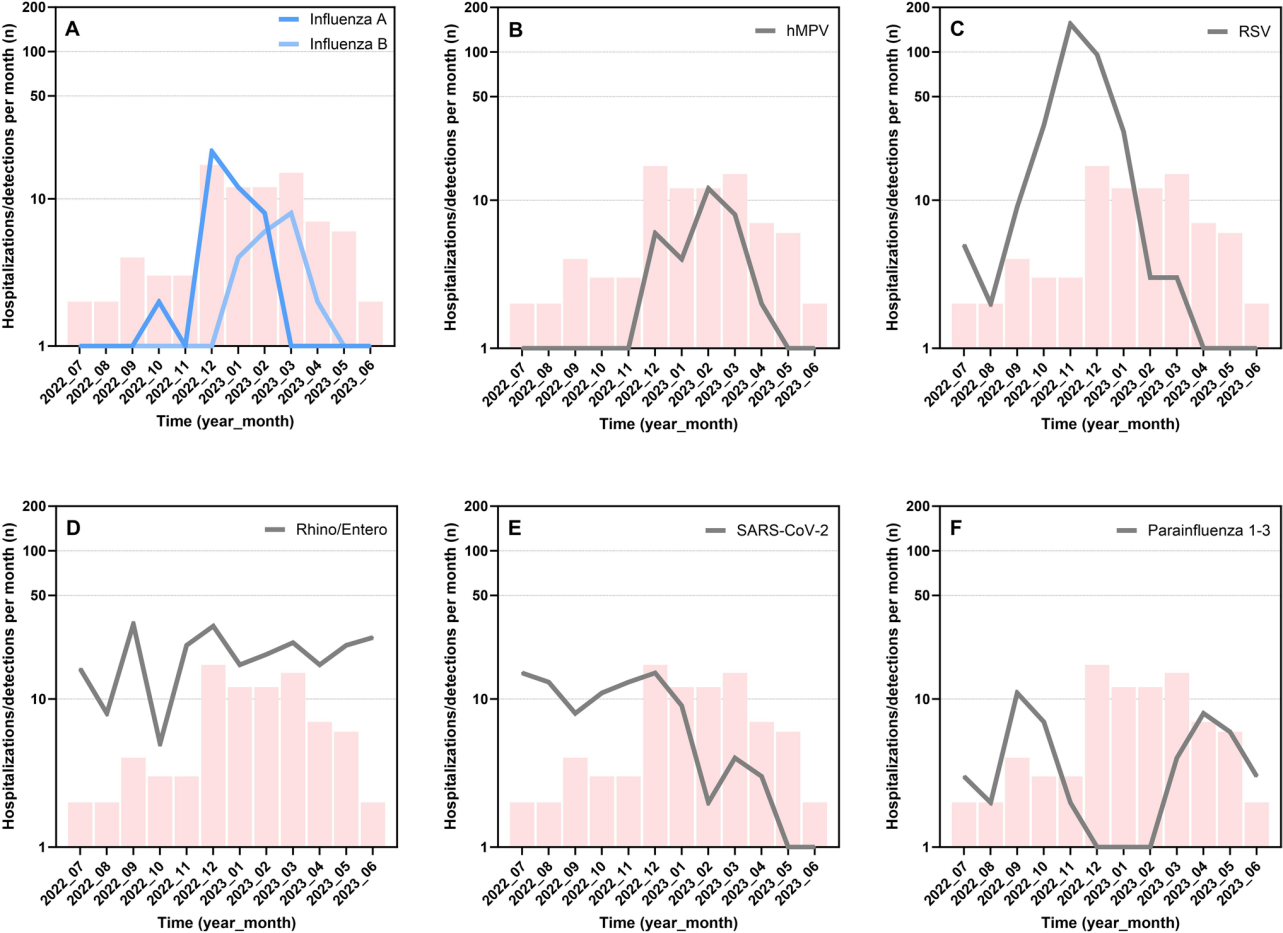
Time series of monthly GAS hospitalization rates (red columns) and monthly detection rates for influenza virus A and B (panel **A**), hMPV (panel **B**), RSV (panel **C**), rhinovirus/enterovirus (panel **D**), SARS-CoV-2 (panel **E**), and parainfluenza virus 1–3 (panel **F**) obtained from the hospital in-patient service between 1 July 2022 and 30 June 2023. The logarithmic scale of the y-axis was chosen to afford better visual comparability of the different panels. Rates of 0 were arbitrarily given the value 1

Figure 3 illustrates the time-series for monthly hospitalization rates in 2022–2023 with GAS and monthly detection rates of common respiratory pathogens. The time course of GAS hospitalizations projects fairly closely over that of influenza, when type A and B cases are combined (Fig. 3, panel A). While the peak of GAS cases in December 2022 coincides with that of influenza A, the protracted decline of GAS cases extending until May 2023 parallels the wave of influenza B from January to April 2023. The time courses of all other viruses examined except hMPV (Fig. 3, panel B) clearly differ from GAS.

## Discussion

The rapid increase of severe GAS infections in children and adolescents in December 2022, which was unexpected in this magnitude, alarmed both primary care and emergency physicians. Many questioned their own outpatient management of patients who were hospitalized for severe GAS infection just days after consultations in their offices, in which they refrained from prescribing an antibiotic. Frequently communicated reasons for non-prescription were (1) that the illness was not suggestive of GAS pharyngitis or (2) that the Swiss recommendations issued in 2019 no longer recommended universal antimicrobial therapy of uncomplicated GAS pharyngitis and scarlet fever [16].

Our findings of the 2022–2023 case cohort in comparison with the cohort admitted from 2013 to 2022 allow us to ease these concerns in two ways. First, the reported prehospitalization illness was suggestive of GAS pharyngitis in significantly fewer patients in 2022–2023 than before. This is indicated by the modified McIsaac score, which in 2022–2023 was consistently lower for three different stringencies (Table 1, Fig. 2, Figure S2). Significantly more patients in 2022–2023 experienced manifestations suggestive of a viral infection, i.e., acute-onset cough and absence of reported cervical lymphadenopathy. Acute nasal symptoms also tended to be more common in this cohort. Unfortunately, we were unable to study true viral co-infections in our patients, because viral tests from nasopharyngeal secretions were not available in the majority of cases. However, we performed a comparison of the 2022–2023 time-series of GAS hospitalizations with respiratory viral detection frequencies in our in-patient service. It revealed a close match with influenza A/B and hMPV, but not RSV, SARS-CoV-2, rhinovirus/enterovirus, or parainfluenza (Fig. 3). We are not aware of studies having examined co-infections of GAS and hMPV. However, the epidemiologic association of severe pediatric GAS disease with influenza outbreaks has been reported before [18–22], although the pathogenesis of this interaction (recently reviewed by Okahashi et al. [23]) is far less well explored than that between *Streptococcus pneumoniae* and influenza. Interactions between GAS and influenza have mainly been reported for influenza A [22]. Of particular interest in our context, however, are observations from the 2010/2011 influenza season in England and France [24, 25], which described the association in time of both severe and non-severe GAS infections and, specifically, influenza B. As reported from other European countries [26], the late 2022–2023 influenza season was dominated by influenza B. In Switzerland, this type was responsible for an unusually prolonged influenza activity lasting until April 2023 [27] and coincided precisely with both the GAS and influenza time courses in our study (Fig. 3). The significant increase in cases of GAS pleural empyema in 2022–2023 compared to 2013–2022, which was also reported from, e.g., Scotland, Denmark, and France [8, 28, 29], also suggests that respiratory viral co-infections played an important role in this outbreak. Interestingly, the French data also suggest a temporal association of GAS pleural empyema cases with the influenza virus activity, while hMPV detections were infrequent and appeared unrelated [8].

Second, we found no evidence supporting our initial hypothesis that in 2022–2023, a greater proportion of patients who presented with a GAS pharyngitis-like prodrome at an outpatient visit were not given an antibiotic prescription than in earlier years. This proportion of withheld antibiotic prescription was small in general, and, surprisingly, smaller still in 2022–2023 than before (Fig. 2). It is possible that the awareness about the current GAS outbreak among primary care physicians favored a more liberal use of antibiotics than stipulated in the new recommendations [13]. In any event, however, the potential impact of wider antibiotic use in preventing hospitalizations, if the old recommendations had still been in place, would have been negligible. It is important to note also that throughout the entire observation period of this study, two thirds of all patients had no physician contact ≥ 1 day before admission. Thus, only a minority of cases would have been amenable to physician-led interventions preventing hospitalization. This finding and, in particular, the fact that in 2022–2023, the duration of preadmission illness was significantly longer than in 2013–2022 (Figure S3) suggests that parents were not particularly worried by their child’s condition during the first few days of the illness, and that clinical deterioration occurred quickly thereafter. Again, such a scenario would fit well the concept that viral symptoms, which are usually not considered worrisome by many parents, dominated the early preadmission period of illness.

A well-established disadvantage of antibiotic non-therapy of GAS pharyngitis is the increased risk for suppurative complications, such as deep pharyngeal abscesses (quinsy) and middle ear disease [9]. Again, in our dataset, we failed to identify major differences in the relative frequency of such cases between the two study periods (Table S2). This is not surprising given the small proportion of patients overall who were exposed to an oral antibiotic early in the course of disease.

We acknowledge several limitations of our methodological approach. The single-center, retrospective design implies that data represent a limited geographic area and entail a limited precision of the reported clinical manifestations, which largely relied on the parents’ recollection and the physicians’ detail in recording. Also, in December 2022, we switched from retrospective to prospective case catchment. However, data extraction from the medical records remained unchanged, and therefore, we do not consider that this has resulted in a bias. Moreover, our modification of the McIsaac Score had not undergone validation in a separate study. It is less precise than the original McIsaac score for 3 out of 5 items (temperature, cervical lymphadenopathy, and tonsillar appearance) and, consequently, overestimates the compatibility with GAS pharyngitis. We attempt to compensate for this by presenting or findings for the scores of ≥ 2, ≥ 3, and ≥ 4. However, greater precision would likely have reduced the proportion of patients presenting with GAS pharyngitis-like manifestations further, which would support the main message of this study. Furthermore, the McIsaac score was devised for patients above 3 years of age only [16], while we included all patients below 16 years of age. We therefore also provided the main findings for patients 3–15 years of age as (Fig. 2C, D and Figure S2, panel B) to demonstrate that the results closely resemble those of the entire cohort. Finally, the cut-off dates for the two cohorts were arbitrarily set and not linked to either the abandonment of most non-pharmaceutical COVID-19 interventions (February 2022) or the implementation of the new management recommendations for uncomplicated GAS pharyngitis. Therefore, we also tested 1 January 2020 instead of 1 July 2022 as an alternative cut-off date and found no relevant changes in the main results of this study (data not shown). Thus, this study provides evidence that (1) respiratory viral infections, notably influenza A and B, may have been an important driver of the extraordinary upsurge of pediatric GAS hospitalizations in 2022–2023, and that (2) only in a negligible proportion of patients could a preadmission outpatient consultation be considered a potentially missed opportunity for an oral antibiotic prescription. The findings should provide reassurance to clinicians for continuing the current practice of using antibiotics sparingly in cases of uncomplicated GAS pharyngitis as stipulated in the current recommendations [13].

### Supplementary Information

Below is the link to the electronic supplementary material.

Supplementary file1 (DOCX 98 KB)

## Data Availability

Anonymized data that support the findings of this study are available on request from the corresponding author, [NS].
