# Supplementary material for: Preadmission course and management of severe pediatric group A streptococcal infections during the 2022–2023 outbreak: a single-center experience
Source: Infection. 2024 Mar 1;52(4):1397–405. doi: 10.1007/s15010-024-02198-w (PMC11289297; doi:10.1007/s15010-024-02198-w)

**Supplementary information**

Journal: Infection

Title:
Preadmission Course and Management of Severe Pediatric Group A Streptococcal Infections during the 2022-2023 Outbreak – a Single Center Experience

Authors:
Nina Schöbi,^1^ Andrea Duppenthaler,^1^ Matthias Horn,^1^ Andreas Bartenstein,^2^ Kristina Keitel,^3^ Matthias V Kopp,^1,4^ Philipp Agyeman,^1^ and Christoph Aebi^1^

Author affiliations:
^1^ Division of Pediatric Infectious Disease, Department of Pediatrics, Bern University Hospital, Inselspital, University of Bern, Switzerland
^2^ Department of Pediatric Surgery, Bern University Hospital, Inselspital, University of Bern, Switzerland
^3^ Pediatric Emergency Center, Department of Pediatrics, Bern University Hospital, Inselspital, University of Bern, Switzerland
^4^ Airway Research Center North (ARCN), Member of the German Lung Research Center (DZL), University of Lübeck, Germany

Correspondence to:
Schöbi Nina, Division of Pediatric Infectious Disease, Department of Pediatrics, Bern University Hospital, Inselspital, University of Bern, CH-3010 Bern, Switzerland
E-mail: nina.schoebi@insel.ch.

Table S1. Modification of the sore throat score devised by McIsaac et al. [ref] for the purpose of the present study

|  | Criteria | |
| --- | --- | --- |
| Points | McIsaac Score | Score adapted for this study |
| 1 | Temperature >38°C | Fever^1^ |
| 1 | No cough | No cough |
| 1 | Tender anterior cervical lymphadenopathy | Cervical lymphadenopathy^2^ |
| 1 | Tonsillar swelling or exsudate | Pharyngitis^3^ |
| 1 | Age 3-14 yr | Age 3-15 yr^4^ |
| 0 | Age 15-44 yr | Age < 3 yr |
| -1 | Age ≥ 45 yr | Not applicable |
| Maximum score | 5 points | 5 points |

Comments
^1^ parental or patient reporting of fever without reported exact temperature was accepted.
^2^ cervical lymphadenopathy did not require reporting of the exact anatomic location and whether there was tenderness because of inconsistent precision in the case notes.
^3^ any of the following written descriptions were acceptable because of inconsistent precision in the case notes: pharyngitis, pharyngeal erythema, tonsillitis, tonsillar erythema, tonsillar swelling, tonsillar exsudate.
^4^ adapted to fit the age range of patients included in this study.

Table S2. List of diagnoses in children and adolescents hospitalized for GAS disease in the cohorts of 2022-2023 vs. 2013-2022

|  | Observation period | | |
| --- | --- | --- | --- |
|  | 2022-2023 | 2013-2022 | 2013-2023 |
| Location | n (iGAS) | n (iGAS) | n (iGAS) |
| *Head, eye, ear, nose, throat* | 45 (5)* | 123 (8) | 168 (13) |
| Peritonsillar/para-/retropharyngeal abscess | 16 (0)** | 53 (1)** | 69 (1) |
| Mastoiditis | 17 (1)*** | 21 (0)*** | 38 (1) |
| Orbital cellulitis/abscess | 4 (2) | 11 (4) | 15 (6) |
| Pharyngitis | 2 (0) | 18 (0) | 20 (0) |
| Cellulitis/abscess in other locations | 6 (2) | 20 (3) | 26 (5) |
|  |  |  |  |
| *Skin and soft tissue* | 14 (3) | 51 (11) | 65 (14) |
| Cellulitis | 9 (1) | 31 (4) | 40 (5) |
| Abscess | 3 (0) | 17 (4) | 20 (4) |
| Necrotizing fasciitis | 2 (2) | 2 (2) | 4 (4) |
| Peritonitis | 0 | 1 (1) | 1 (1) |
|  |  |  |  |
| *Respiratory tract* | 13 (12) | 12 (10) | 25 (22) |
| Pleural empyema | 11 (11) | 11 (10) | 22 (21) |
| Pneumonia | 2 (1) | 0 | 2 (1) |
| Tracheitis | 0 | 1 (0) | 1 (0) |
|  |  |  |  |
| *Skeletal system* | 10 (10) | 6 (6) | 16 (16) |
| Osteomyelitis | 5 (5) | 4 (4) | 9 (9) |
| Myositis | 3 (3) | 1 (1) | 4 (4) |
| Arthritis | 2 (2) | 1 (1) | 3 (3) |
|  |  |  |  |
| *Central nervous system* | 0 | 2 (2) | 2 (2) |
| Meningitis | 0 | 1 (1) | 1 (1) |
| Brain abscess | 0 | 1 (1) | 1 (1) |
|  |  |  |  |
| *Systemic onset (no primary site identified)* | 3 (2) | 6 (6) | 9 (8) |
| bacteremia | 2 (2) | 2 (2) | 4 (4) |
| septic/toxic shock | 0 | 4 (4) | 4 (4) |
| lobar nephronia | 1 (0) | 0 | 1 (0) |
|  |  |  |  |
| *Total* | 85 (32) | 200 (43) | 285 (75) |
|  |  |  |  |
| * throughout the table the numbers in parentheses indicate the number of iGAS cases  ** OR 0.64 (95% CI 0.34-1.21, p=0.166) | | | |
| *** OR 2.13 (95% CI 1.06-4.28; p=0.031) |  |  |  |

Figure S1. Age distribution in children and adolescents hospitalized for GAS disease in the cohorts of 2022-2023 (red) vs. 2013-2022 (blue).


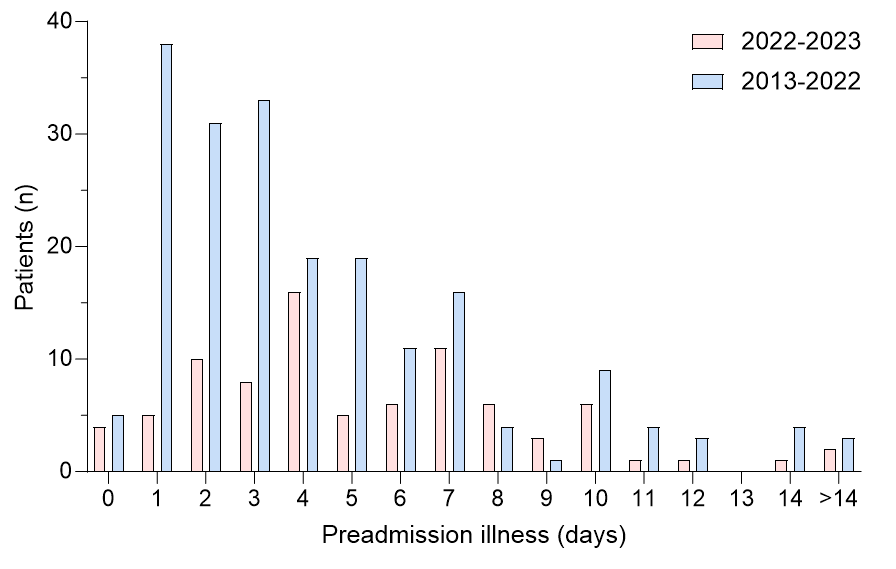


Figure S2. Distribution of modified McIsaac Scores in the cohorts of 2022-2023 (red) vs. 2013-2022 (blue). Panel A, all patients; Panel B, patients ≥3 years of age


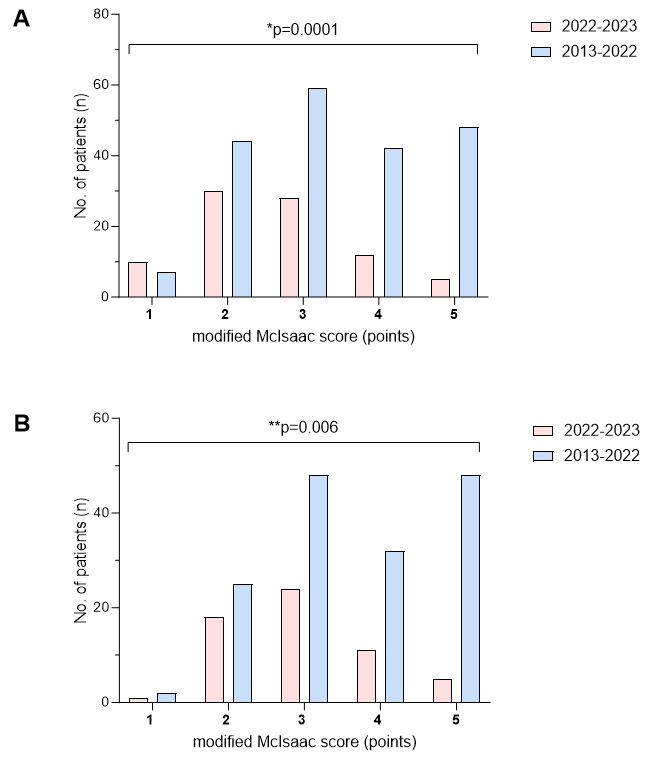


*Χ^2^ test für 2x5 contingency table (Cramer’s V = 0.285)

** Χ^2^ test für 2x5 contingency table (Cramer’s V = 0.259)

Figure S3. Distribution of the duration of preadmission illness (days) in patients hospitalized for GAS disease in 2022-2023 (red) vs. 2013-2022 (blue)


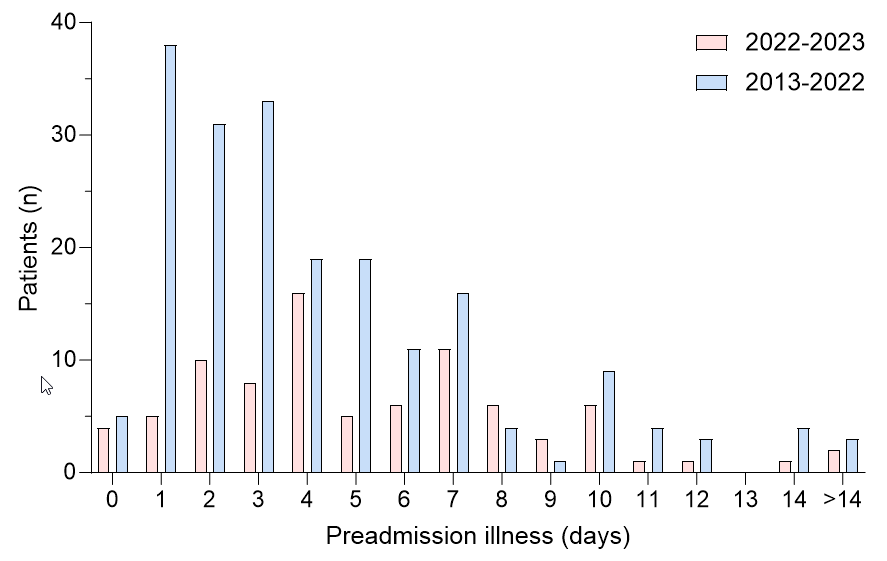

Supplement: Supplementary file 1 — Supplementary file1 (DOCX 98 KB) [file 15010_2024_2198_MOESM1_ESM.docx]
